# Publisher Correction: The Interplay between Incipient Species and Social Polymorphism in the Desert Ant *Cataglyphis*

**DOI:** 10.1038/s41598-020-57950-7

**Published:** 2020-02-04

**Authors:** Tali Reiner Brodetzki, Shani Inbar, Pnina Cohen, Serge Aron, Eyal Privman, Abraham Hefetz

**Affiliations:** 1 School of Zoology, George S. Wise Faculty of Life Sciences, Tel Aviv University, Tel Aviv, 6997801 Israel; 2 Institute of Evolution, Department of Evolutionary and Environmental Biology, University of Haifa, Haifa, Israel; 3 Evolutionary Biology and Ecology, Université Libre de Bruxelles, Université D’Europe, Brussels, Belgium

Correction to: *Scientific Reports* 10.1038/s41598-019-45950-1, published online 01 July 2019

In the original version of this Article, the author Tali Reiner Brodetzki was incorrectly indexed. This error has now been corrected.

