# Supplementary material for: The Interplay between Incipient Species and Social Polymorphism in the Desert Ant Cataglyphis
Source: Sci Rep. 2019 Jul 1;9:9495. doi: 10.1038/s41598-019-45950-1 (PMC6603034; doi:10.1038/s41598-019-45950-1)
Supplement: Supplementary file 1 — Supplementary information [file 41598_2019_45950_MOESM1_ESM.pdf]

Supplementary information

The Interplay between **Incipient Species and Social Polymorphism in the Desert Ant *Cataglyphis***

Tali Reiner Brodetzki[1], Shani Inbar[2], Pnina Cohen[2], Serge Aron[3], Eyal Privman[2], and Abraham Hefetz[1]

[1] School of Zoology, George S. Wise Faculty of Life Sciences, Tel Aviv University, Tel Aviv, 6997801, Israel.

[2] Institute of Evolution, Department of Evolutionary and Environmental Biology, University of Haifa, Israel.

[3] Evolutionary Biology and Ecology, Université Libre de Bruxelles, Université D'Europe

Supplementary figures captions:

Figure S1: Sampling map of sample location of ants for the ddRAD. Locations are colored acourding to their genetic cluster.

Figure S2: comparison of reads coverage of each ant at the stage of raw reads (red) and after the alignment-based filtering (blue). Notice that the initial coverage is based on averaging the number of reads over the known number of restriction sites, while the final coverage is based on actual mapping of the reads to the reference genome of *C. drusus* and is therefore more accurate.

Figure S3: A) Typical cuticular hydrocarbon profile of the *drusus* mitotype from Betzet. The chromatogram shows only the 60 identified compounds (numbered peaks), of which 35 hydrocarbons were used in the analysis (marked in bold). B) The cuticular hydrocarbon profile from the Tel Baruch population CHC's used for the analysis is marked in bold (44 out of 74 identified).

Figure S4: Bayesian inference tree CytB haplotypes of sperm content from *niger* queens. Bayesian probabilities/bootstrap values (from Ugene) are given to estimate branch support. The trees are rooted using the haplotype sequences from Eyer *et al*. (2017).

Figure S1

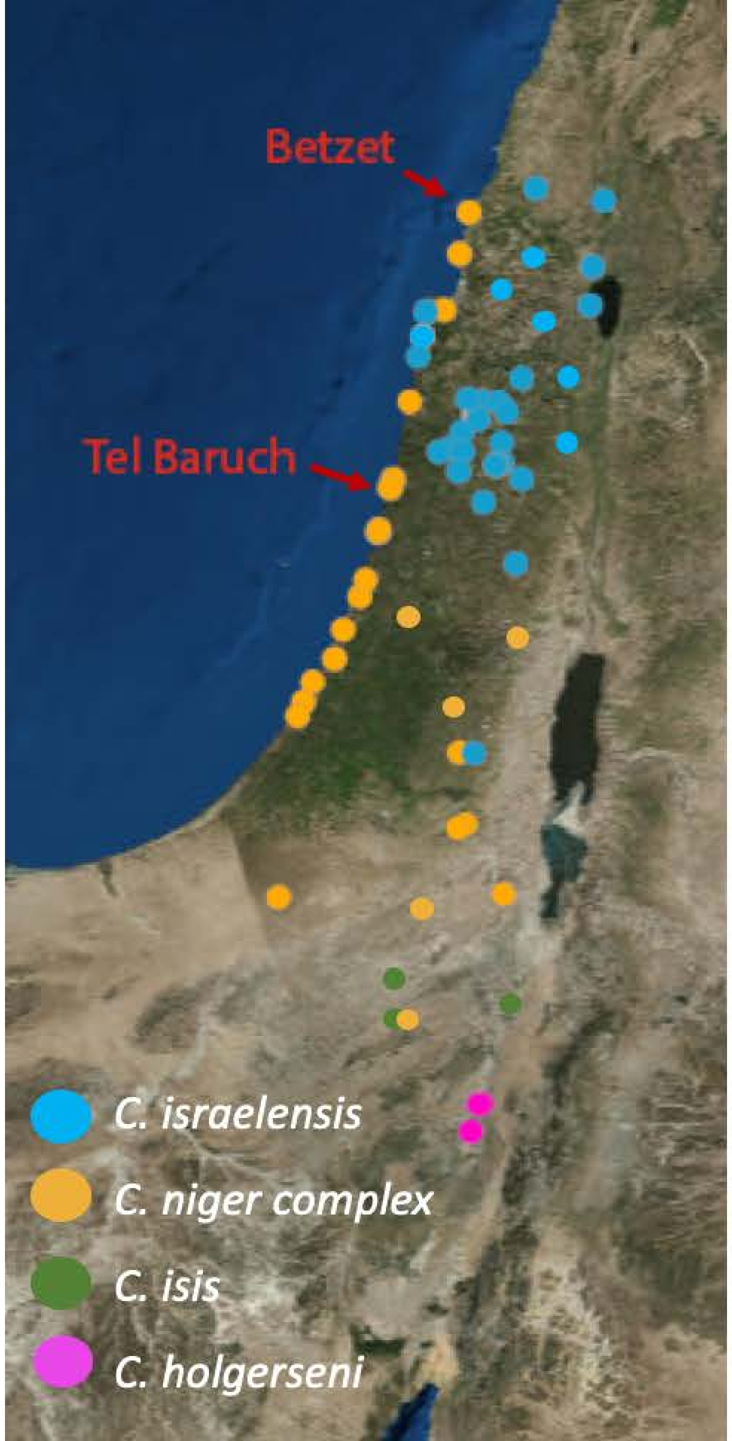

Map was created using ArcGIS® software by Esri. ArcGIS® and ArcMap™ are the intellectual property of Esri and are used herein under license. Copyright © Esri. All rights reserved. For more '.information about Esri® software, please visit www.esri.com

Figure S2

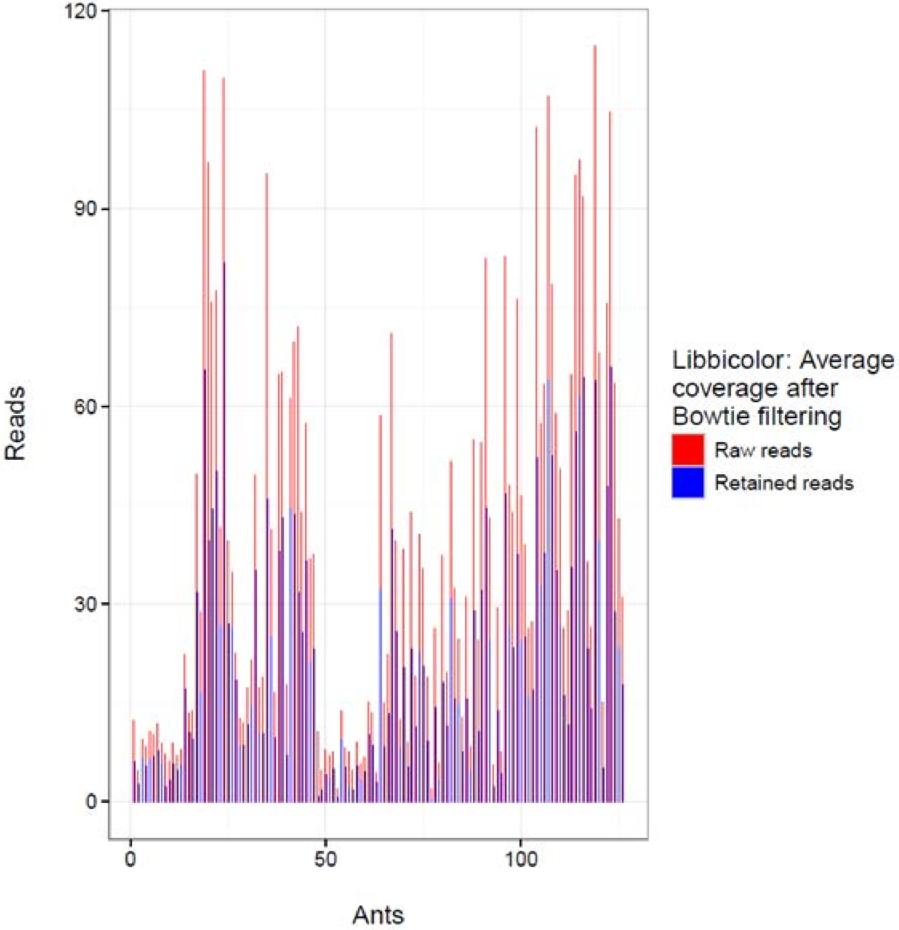

120
90
60
30
0
Reads
0
50
100
Ants
Libbicolor: Average coverage after Bowtie filtering
Raw reads
Retained reads

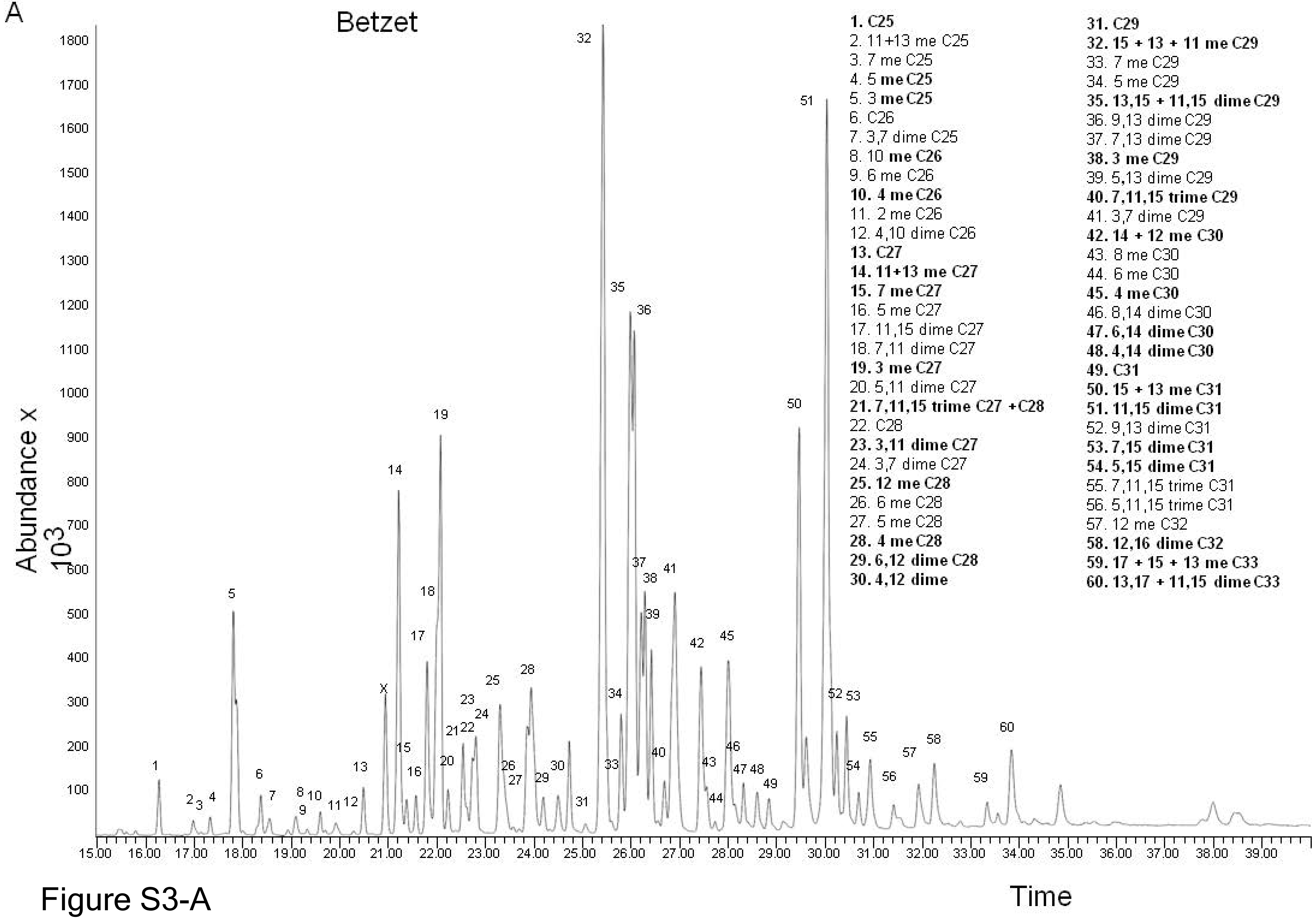
A
Betzet
Abundance X 10³
1800
1700
1600
1500
1400
1300
1200
1100
1000
900
800
700
600
500
400
300
200
100
1. C25
2. 11+13 me C25
3. 7 me C25
4. 5 me C25
5. 3 me C25
6. C26
7. 3,7 dime C25
8. 10 me C26
9. 6 me C26
10. 4 me C26
11. 2 me C26
12. 4,10 dime C26
13. C27
14. 11+13 me C27
15. 7 me C27
16. 5 me C27
17. 11,15 dime C27
18. 7,11 dime C27
19. 3 me C27
20. 5,11 dime C27
21. 7,11,15 trime C27 +C28
22. C28
23. 3,11 dime C27
24. 3,7 dime C27
25. 12 me C28
26. 6 me C28
27. 5 me C28
28. 4 me C28
29. 6,12 dime C28
30. 4,12 dime
31. C29
32. 15 + 13 + 11 me C29
33. 7 me C29
34. 5 me C29
35. 13,15 + 11,15 dime C29
36. 9,13 dime C29
37. 7,13 dime C29
38. 3 me C29
39. 5,13 dime C29
40. 7,11,15 trime C29
41. 3,7 dime C29
42. 14 + 12 me C30
43. 8 me C30
44. 6 me C30
45. 4 me C30
46. 8,14 dime C30
47. 6,14 dime C30
48. 4,14 dime C30
49. C31
50. 15 + 13 me C31
51. 11,15 dime C31
52. 9,13 dime C31
53. 7,15 dime C31
54. 5,15 dime C31
55. 7,11,15 trime C31
56. 5,11,15 trime C31
57. 12 me C32
58. 12,16 dime C32
59. 17 + 15 + 13 me C33
60. 13,17 + 11,15 dime C33
15.00
16.00
17.00
18.00
19.00
20.00
21.00
22.00
23.00
24.00
25.00
26.00
27.00
28.00
29.00
30.00
31.00
32.00
33.00
34.00
35.00
36.00
37.00
38.00
39.00
Time

Figure S3-A

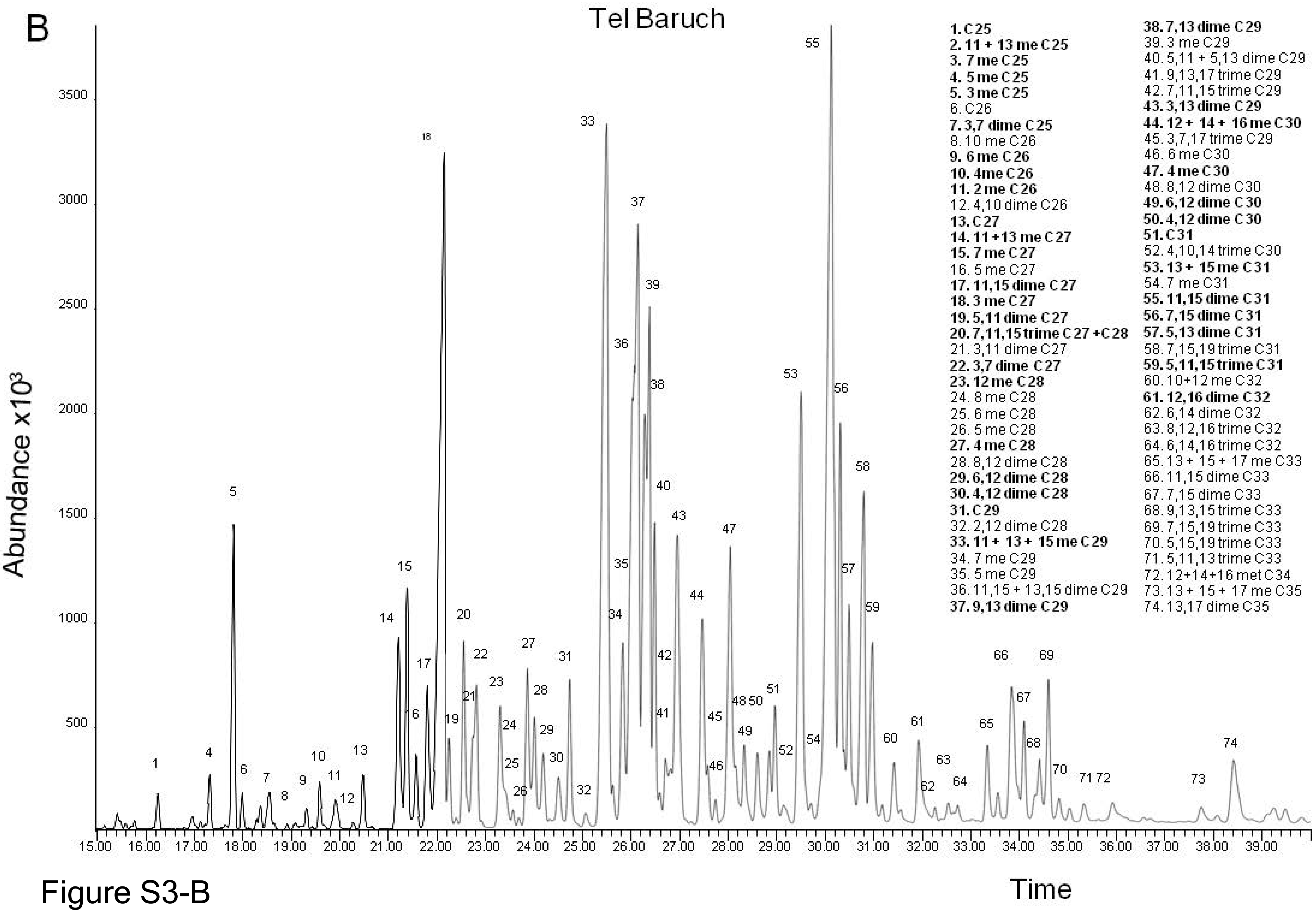
B
Tel Baruch
Abundance x10³
Time
1. C25
2. 11 + 13 me C25
3. 7 me C25
4. 5 me C25
5. 3 me C25
6. C26
7. 3,7 dime C25
8. 10 me C26
9. 6 me C26
10. 4me C26
11. 2 me C26
12. 4,10 dime C26
13. C27
14. 11+13 me C27
15. 7 me C27
16. 5 me C27
17. 11,15 dime C27
18. 3 me C27
19. 5,11 dime C27
20. 7,11,15 trime C27 +C28
21. 3,11 dime C27
22. 3,7 dime C27
23. 12 me C28
24. 8 me C28
25. 6 me C28
26. 5 me C28
27. 4 me C28
28. 8,12 dime C28
29. 6,12 dime C28
30. 4,12 dime C28
31. C29
32. 2,12 dime C28
33. 11 + 13 + 15 me C29
34. 7 me C29
35. 5 me C29
36. 11,15 + 13,15 dime C29
37. 9,13 dime C29
38. 7,13 dime C29
39. 3 me C29
40. 5,11 + 5,13 dime C29
41. 9,13,17 trime C29
42. 7,11,15 trime C29
43. 3,13 dime C29
44. 12 + 14 + 16 me C30
45. 3,7,17 trime C29
46. 6 me C30
47. 4 me C30
48. 8,12 dime C30
49. 6,12 dime C30
50. 4,12 dime C30
51. C31
52. 4,10,14 trime C30
53. 13 + 15 me C31
54. 7 me C31
55. 11,15 dime C31
56. 7,15 dime C31
57. 5,13 dime C31
58. 7,15,19 trime C31
59. 5,11,15 trime C31
60. 10+12 me C32
61. 12,16 dime C32
62. 6,14 dime C32
63. 8,12,16 trime C32
64. 6,14,16 trime C32
65. 13 + 15 + 17 me C33
66. 11,15 dime C33
67. 7,15 dime C33
68. 9,13,15 trime C33
69. 7,15,19 trime C33
70. 5,15,19 trime C33
71. 5,11,13 trime C33
72. 12+14+16 met C34
73. 13 + 15 + 17 me C35
74. 13,17 dime C35

Figure S3-B

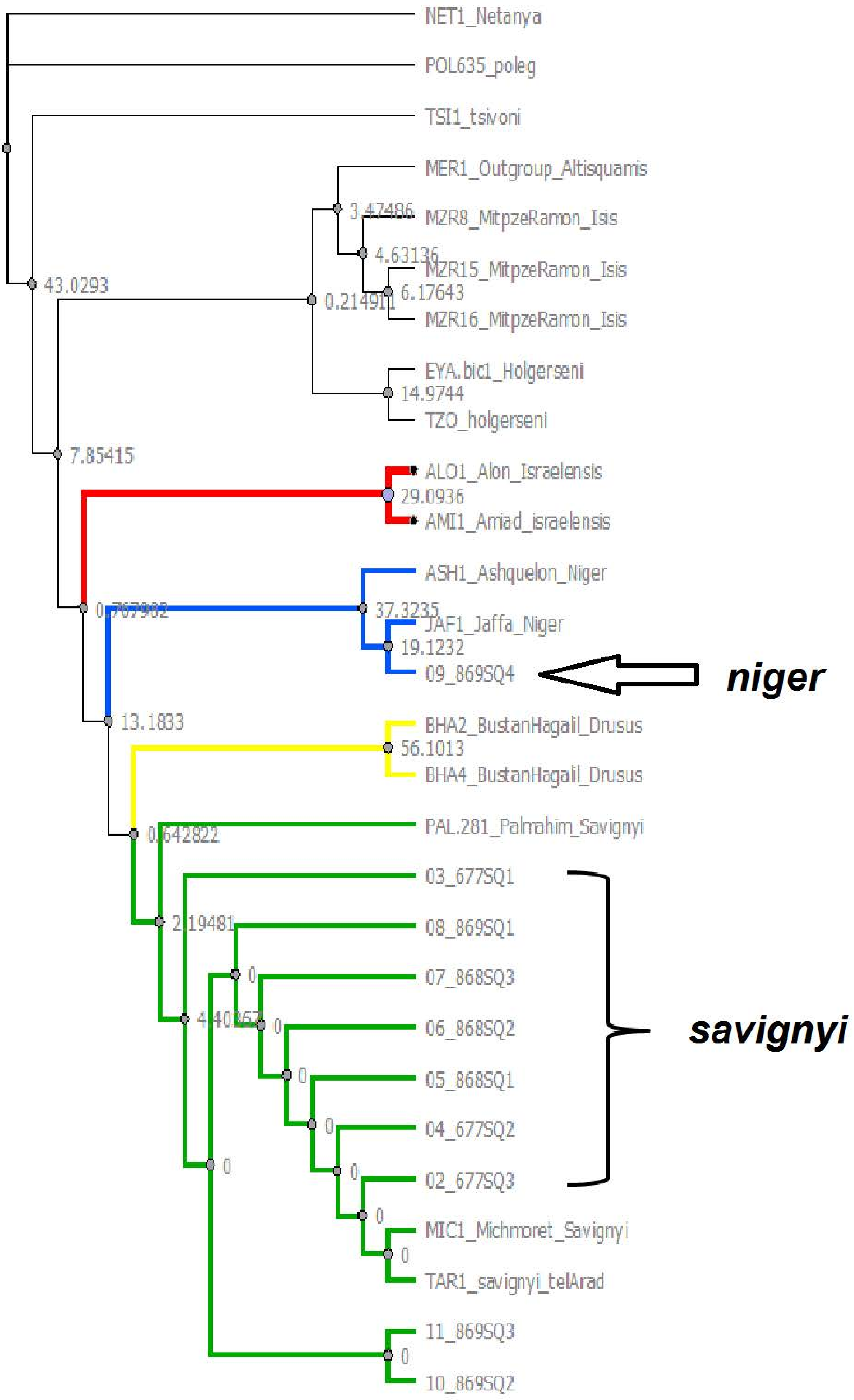
NET1_Netanya
POL635_poleg
TSI1_tsivoni
MER1_Outgroup_Altisquamis
3.47486
MZR8_MitpzeRamon_Isis
4.63136
MZR15_MitpzeRamon_Isis
6.17643
0.214911
MZR16_MitpzeRamon_Isis
43.0293
EYA.bic1_Holgerseni
14.9744
TZO_holgerseni
7.85415
ALO1_Alon_Israelensis
29.0936
AMI1_Amiad_israelensis
ASH1_Ashquelon_Niger
0.767902
37.3235
JAF1_Jaffa_Niger
19.1232
09_869SQ4
niger
13.1833
BHA2_BustanHagalil_Drusus
56.1013
BHA4_BustanHagalil_Drusus
PAL.281_Palmahim_Savignyi
0.642822
03_677SQ1
2.19481
08_869SQ1
07_868SQ3
4.40267
06_868SQ2
savignyi
05_868SQ1
04_677SQ2
02_677SQ3
MIC1_Michmoret_Savignyi
TAR1_savignyi_telArad
11_869SQ3
10_869SQ2

Figure S4

Table 3.S1: GPS coordinates of samples used in the study.

	**Betzet population**		**Tel Baruch population**		
	525	N33 04.651 E35 06.539	454	N32 07.637 E34 47.269	
	526	N33 04.651 E35 06.537	455	N32 07.624 E34 47.288	
	527	N33 04.650 E35 06.536	457	N32 07.606 E34 47.270	
	528	N33 04.675 E35 06.582	461	N32 07.634 E34 47.242	
	530	N33 04.601 E35 06.534	462	N32 07.592 E34 47.192	
	531	N33 04.615 E35 06.522	464	N32 07.613 E34 47.208	
	532	N33 04.619 E35 06.526	465	N32 07.601 E34 47.213	
	533	N33 04.586 E35 06.507	466	N32 07.604 E34 47.217	
	534	N33 04.612 E35 06.545	467	N32 07.595 E34 47.211	
	535	N33 04.600 E35 06.540	469	N32 07.572 E34 47.248	
	538	N33 04.584 E35 06.541	473	N32 07.577 E34 47.254	
	539	N33 04.635 E35 06.510	481	N32 07.592 E34 47.204	
	540	N33 04.658 E35 06.502	487	N32 07.628 E34 47.215	
	541	N33 04.561 E35 06.507	489	N32 07.620 E34 47.198	
	542	N33 04.563 E35 06.509	491	N32 07.601 E34 47.186	
	543	N33 04.559 E35 06.521	498	N32 07.578 E34 47.188	
	544	N33 04.579 E35 06.536	499	N32 07.561 E34 47.154	
	545	N33 04.582 E35 06.525	500	N32 07.562 E34 47.151	
	546	N33 04.585 E35 06.526	505	N32 07.573 E34 47.164	
	547	N33 04.585 E35 06.526	506	N32 07.572 E34 47.162	
	548	N33 04.589 E35 06.523	599	N32 07.616 E34 47.254	
	549	N33 04.567 E35 06.495	600	N32 07.582 E34 47.258	
	550	N33 04.572 E35 06.513	602	N32 07.559 E34 47.161	
	551	N33 04.585 E35 06.513	603	N32 07.557 E34 47.238	
	552	N33 04.665 E35 06.483	604	N32 07.558 E34 47.235	
	553	N33 04.674 E35 06.560	606	N32 07.554 E34 47.243	
	554	N33 04.672 E35 06.562	608	N32 07.616 E34 47.186	
	555	N33 04.678 E35 06.449	640	N32 16.934 E34 50.419	
	556	N33 04.678 E35 06.449	641	N32 07.454 E34 47.125	
	557	N33 04.679 E35 06.448	642	N32 07.455 E34 47.116	
	559	N33 04.671 E35 06.450	643	N32 07.512 E34 47.146	
	560	N33 04.627 E35 06.455	644	N32 07.496 E34 47.162	
	562	N33 04.628 E35 06.460	645	N32 07.537 E34 47.124	
	563	N33 04.652 E35 06.543	648	N32 07.682 E34 47.294	
	564	N33 04.635 E35 06.554	650	N32 07.681 E34 47.281	
	565	N33 04.634 E35 06.557	653	N32 07.951 E34 47.432	
	568	N33 04.586 E35 06.485	654	N32 07.933 E34 47.420	
	570	N33 04.654 E35 06.533	655	N32 07.944 E34 47.416	
	571	N33 04.653 E35 06.534	657	N32 07.770 E34 47.309	
	575	N33 04.595 E35 06.404	658	N32 07.775 E34 47.292	
	576	N33 04.604 E35 06.405	**Merging samples-Betzet**		
	577	N33 04.601 E35 06.405	2	969	N33 04.598 E35 06.566
	578	N33 04.511 E35 06.401	3	970	N33 04.598 E35 06.567
	579	N33 04.507 E35 06.406	1	971	N33 04.608 E35 06.554
	580	N33 04.445 E35 06.379	4	973	N33 04.609 E35 06.549
	581	N33 04.440 E35 06.412	5	974	N33 04.610 E35 06.549
	582	N33 04.366 E35 06.377	7	976	N33 04.600 E35 06.542
	583	N33 04.370 E35 06.392	6	977	N33 04.607 E35 06.549
	584	N33 04.373 E35 06.392	8	979	N33 04.585 E35 06.545
	585	N33 04.323 E35 06.370	9	981	N33 04.607 E35 06.554
	586	N33 04.328 E35 06.375	10	982	N33 04.618 E35 06.561
	587	N33 04.269 E35 06.372	11	983	N33 04.618 E35 06.560
	588	N33 04.267 E35 06.379	13	985	N33 04.583 E35 06.537
	589	N33 04.175 E35 06.358	12	986	N33 04.594 E35 06.540
	590	N33 04.196 E35 06.352	16	987	N33 04.566 E35 06.499
	595	N33 04.097 E35 06.315	15	988	N33 04.564 E35 06.524
	596	N33 04.088 E35 06.318	14	989	N33 04.563 E35 06.524
			17	990	N33 04.565 E35 06.493
			18	991	N33 04.564 E35 06.501

Sample name	Mitotype	Locaion	North	East	Collected by
11	Drusus	Betzet	N334.633	E356.509	TRB
21	Drusus	Bostan hagalil	N3257.208	E354.514	TRB
31	Drusus	Kiryat haim	N3257.277	E354.529	TRB
46	Israelensis	Hof hacarmel	N3246.951	E3457.358	TRB
51	Savignyi	Zikim	N3136.807	E3431.365	TRB
61	Niger	Zikim west	N3136.812	E3431.303	TRB
76	Savignyi	Askelon	N3140.471	E3433.256	TRB
86	complex	Nitzan	N3144.647	E3437.954	TRB
100	complex	Nitzan	N3144.673	E3437.92	TRB
116	Savignyi	Palmachim	N3155.92	E3443.322	TRB
146	Savignyi	Rishon	N3158.874	E3444.517	TRB
211	Israelensis	Tyrat carmel	N3246.791	E3457.334	TRB
241	Savignyi	Ceseria	N3230.84	E3453.892	TRB
454	Niger	Tel Baruch	N327.637	E3447.269	TRB
461	Niger	Tel Baruch	N327.634	E3447.242	TRB
464	savignyi	Tel Baruch	N327.613	E3447.208	TRB
465	complex	Tel Baruch	N327.601	E3447.213	TRB
469	Savignyi	Tel Baruch	N327.572	E3447.248	TRB
481	savignyi	Tel Baruch	N327.592	E3447.204	TRB
486	niger	Tel Baruch	N327.629	E3447.216	TRB
487	niger	Tel Baruch	N327.628	E3447.215	TRB
489	savignyi	Tel Baruch	N327.62	E3447.198	TRB
491	niger	Tel Baruch	N327.601	E3447.186	TRB
499	savignyi	Tel Baruch	N327.561	E3447.154	TRB
500	Savignyi	Tel Baruch	N327.562	E3447.151	TRB
505	Savignyi	Tel Baruch	N327.573	E3447.164	TRB
600	savignyi	Tel Baruch	N327.582	E3447.258	TRB
602	savignyi	Tel Baruch	N327.559	E3447.161	TRB
608	Niger	Tel Baruch	N327.616	E3447.186	TRB
641	Drusus	Tel Baruch	N327.454	E3447.125	TRB
642	Drusus	Tel Baruch	N327.455	E3447.116	TRB
643	Savignyi	Tel Baruch	N327.512	E3447.146	TRB
644	Drusus	Tel Baruch	N327.496	E3447.162	TRB
650	Niger	Tel Baruch	N327.681	E3447.281	TRB
653	Niger	Tel Baruch	N327.951	E3447.432	TRB
657	complex	Tel Baruch	N327.77	E3447.309	TRB
922	Israelensis	Atlit	N3242.587	E3456.623	TRB
993	Israelensis	Tveria	N3248.044	E3532.211	TRB
994	Israelensis	Tveria	N3248.021	E3532.223	TRB
995	Israelensis	Amiad	N3254.951	E3532.76	TRB
996	Israelensis	Amiad	N3254.952	E3532.761	TRB
997	Israelensis	Agmon	N336.708	E3535.079	TRB
999	Savignyi	Vingate	N3215.381	E3449.7	TRB
1000	Savignyi	Vingate	N3215.381	E3449.7	TRB
1001	Savignyi	Vingate	N3215.381	E3449.7	TRB
498	Savignyi	Tel Baruch	N327.578	E3447.188	TRB
506	Savignyi	Tel Baruch	N327.572	E3447.162	TRB
641tb	Drusus	Tel Baruch	N327.454	E3447.125	TRB
642tb	Drusus	Tel Baruch	N327.455	E3447.116	TRB
885hab	Israelensis	Habonim	N3238.932	E3455.664	TRB
anm1	Israelensis	Allonim	32°42'40.2"N	35°08'33.9"E	Eyer et al. 2017
ash101	Savignyi	Ashdod	N3149.883	E3439.777	Eyer et al. 2017
asv1	Israelensis	Azmon Segev	32°51'47,6"N	35°15'13,1"E	Eyer et al. 2017
bim1	Israelensis	Bir El Maksur	32°46'05,5"N	35°14'08,2"E	Eyer et al. 2017
bmh3	Isis	Be'er Menuha	30°18'3.71"N	35° 7'53.97"E	Eyer et al. 2017
dha1	Israelensis	Deir Hanna	32°51'35.7"N	35°21'35.7"E	Eyer et al. 2017
dov3	Israelensis	Daverat	32°39'14.2"N	35°22'39.7"E	Eyer et al. 2017
eha1	Israelensis	En Harod (Me'uhad)	32°33'21.6"N	35°22'59.3"E	Eyer et al. 2017
ehn4	Israelensis	En HaNaziv	32°28'36.4"N	35°29'54.6"E	Eyer et al. 2017
ely3	Israelensis	Elyaqim	32°37'51,2"N	35°04'10,4"E	Eyer et al. 2017
evd	Isis	En 'Avedat	30°50'49.99"N	34°46'45.46"E	Eyer et al. 2017
eya1	Holgerseni	En Yahav 2	30°39'51.32"N	35°15'36.72"E	Eyer et al. 2017
eyh1	Holgerseni	En Yahav	30°39'44.79"N	35°14'19.59"E	Eyer et al. 2017
gaa3	Savignyi	Ga'ash	32°14'25,50"N	34°49'44,63"E	Eyer et al. 2017
ghs	Savignyi	Gan hashomron	32°28'04,3"N	34°59'25,2"E	Eyer et al. 2017
gid1	Israelensis	Gid'ona	32°32'48,3"N	35°21'30,2"E	Eyer et al. 2017
hbk1	Israelensis	Ha---Biq'a	32°03'01.7"N	35°27'44.2"E	Eyer et al. 2017
her1	Savignyi	Hertzelia	32°09'45.36"N	34°48'03.07"E	TRB
her2	Savignyi	Hertzelia	32°09'45.36"N	34°48'03.07"E	TRB
hoa3	Israelensis	Horbat 'Ammudim	32°48'46,4"N	35°24'25,3"E	Eyer et al. 2017
hzy1	Israelensis	HaZayit	32°36'44,8"N	35°00'20,6"E	Eyer et al. 2017
hzy1_2	Niger	Jaffa	32°36'44,8"N	35°00'20,6"E	Eyer et al. 2017
jaf1	Niger	Jaffa	32°2435,91"N	34°44'48,63"E	Eyer et al. 2017
jaf2	Niger	Jaffa	32°2435,91"N	34°44'48,63"E	Eyer et al. 2017
jer1	Israelensis	Jerusalem	31°45'8.94"N	35°12'50.50"E	Eyer et al. 2021
maaon	unknown	Ma'aon	31°24'52.53"N	35°09'50.01"E	Friedman L.
mae1	Israelensis	Ma'ale Efrayim	32°03'58,6"N	35°23'59,0"E	Eyer et al. 2017
mhe	Israelensis	Mishmar Ha'Emeq	32°36'47,1"N	35°08'50,3"E	Eyer et al. 2017
mic1	Savignyi	Michmoret	32°23'51.50"N	34°52'13.60"E	TRB
mic2	Savignyi	Michmoret	32°23'51.50"N	34°52'13.60"E	TRB
mzr1	unknown	Mizpe Ramon	30°36'18.11"N	34°51'57.24"E	Rozenberg T.
mzr3_2	unknown	Mizpe Ramon	30°36'18.11"N	34°51'57.24"E	Rozenberg T.
mzr4	unknown	Mizpe Ramon	30°36'18.11"N	34°51'57.24"E	Rozenberg T.
NEG2	Isis	Negev point 880	30°18'0.08"N	34°58'6.16"E	Eyer et al. 2017
NET3	Savignyi	Netanya	32°18'40,2"N	34°50'43,2"E	Eyer et al. 2017
ngv3	unknown	Negev point 881	30°26'25.76"N	34°56'19.55"E	Eyer et al. 2017
pol633	savignyi	Poleg	N3247.218	E351.229	TRB
pol634	savignyi	Poleg	N3216.877	E3450.31	TRB
pol635	savignyi	Poleg	N3216.906	E3450.333	TRB
pol636	savignyi	Poleg	N3216.897	E3450.334	TRB
pol637	savignyi	Poleg	N3216.915	E3450.417	TRB
poleg638	savignyi	Poleg	N3216.933	E3450.42	TRB
qed	Israelensis	Qedumim	32.212896	35.159068	Friedman L.
revpa6	Savignyi	Revivim	31°02'39.2"N	34°43'34.0"E	Eyer PA
sat	Savignyi	Sattaf	31°46'18.6"N	35°07'38.9"E	Eyer et al. 2017
sdo1	Savignyi	Sedom	31°03'27,26"N	35°23'02,13"E	Renan I.
sus	Savignyi	Susya	31°23'39,07"N	35°07'01,00"E	Friedman L.
taf	Savignyi	tel Afek	32° 7'32.43"N	34°53'44.21"E	Eyer et al. 2017
tam2	Israelensis	Tamra	32°52'31,2"N	35°10'33,7"E	Eyer et al. 2017

Table S2: The number of nests used in each of the analysis.

Location	QL nests	QR nests	Nests in merging experiment	Nests in microsat analysis	Nests in CHC analysis
Betzet	76	5	22	57	49
Tel Baruch	39	21	10	26	28

Table S3: Assumed matrilines according to COLONY in the Betzet population.

Nest #	Cn02		Cn02		Ch08		Cc54		Ch12		Ch01		Ch11		Ch10	
543	90	90	105	113	130	136	213	213	93	97	176	194	235	235	274	304
550	90	90	105	113	130	136	213	213	93	97	176	194	235	247	274	304
579	90	90	109	113	136	136	207	217	117	117	174	180	233	235	304	310
578	90	90	113	113	136	136	207	217	117	117	174	178	233	235	304	310
533	90	90	111	111	128	130	209	219	85	103	176	176	235	249	304	310
551	90	90	111	111	128	130	209	219	85	103	176	176	235	249	304	310
565	90	90	111	111	128	130	207	213	85	85	176	180	245	249	298	298
564	90	90	111	111	128	130	207	213	85	85	176	180	245	249	298	298
525	90	90	111	113	130	132	213	219	0	0	174	178	233	235	286	298
527	90	90	111	113	130	132	213	219	0	0	174	178	233	235	286	298
570	90	90	111	113	130	132	213	219	0	0	174	174	233	235	286	298
577	90	90	111	113	128	136	209	217	93	117	174	176	233	235	262	298
576	90	90	111	113	128	136	209	217	93	117	174	176	233	235	262	298
535	90	90	109	111	134	136	211	219	93	117	174	176	247	249	262	306
530	90	90	109	111	134	136	211	219	93	117	174	176	247	249	262	306
560	90	90	111	119	128	136	209	217	85	85	176	178	233	245	286	304
562	90	90	111	119	128	136	209	209	85	85	176	178	233	245	286	304
544	90	107	113	119	128	130	209	213	0	0	174	178	247	247	286	304
538	90	107	113	119	128	130	209	213	0	0	174	178	247	247	286	304
545	90	107	111	111	130	136	213	219	85	103	176	178	247	251	274	314
546	90	107	111	111	130	136	213	219	0	0	176	178	247	251	274	314
547	90	107	111	111	130	136	213	219	0	0	176	178	247	251	274	314
548	90	107	111	111	130	136	213	219	0	0	176	178	247	251	274	314
581	90	90	105	113	130	130	209	211	93	103	174	176	235	245	286	298
589	90	90	105	113	128	136	213	217	85	117	174	219	233	233	286	298
563	90	90	111	111	130	132	213	219	0	0	174	178	233	247	286	298
532	90	90	111	113	136	136	209	217	0	0	174	174	233	249	286	298
554	90	90	113	119	130	136	209	217	0	0	174	178	235	235	298	298
531	90	90	105	113	128	136	209	217	93	97	174	176	233	249	286	298
549	90	90	111	119	128	136	207	217	0	0	174	176	249	249	286	298
542	90	90	105	113	130	136	209	213	93	97	176	194	235	249	274	304
585	90	100	111	113	130	134	207	219	93	97	176	180	233	235	298	314
588	90	107	111	119	128	128	209	213	0	0	174	174	233	247	298	314
586	90	90	113	119	130	136	211	213	87	117	176	176	233	247	274	298
580	90	90	109	113	130	134	207	209	85	85	174	196	235	235	274	314
568	90	90	111	111	134	136	211	215	85	97	174	176	233	245	274	298
571	90	90	111	113	132	136	213	219	85	93	174	176	233	235	286	298
526	90	90	111	113	130	132	213	219	85	85	174	190	233	235	286	298
559	90	107	109	113	130	130	209	209	0	0	174	178	235	245	298	304
557	90	90	111	119	130	136	209	217	85	83	174	178	233	247	298	304
555	90	90	111	119	130	136	209	217	0	0	174	178	233	249	298	304
556	90	90	111	119	130	136	209	217	0	0	174	176	233	249	298	304
528	88	90	105	109	128	134	207	211	87	117	174	176	247	251	298	298
575	90	90	111	113	128	136	209	217	0	0	174	176	233	235	262	298
553	90	90	113	119	130	136	209	209	0	0	180	196	235	247	298	298
587	90	90	113	119	134	136	209	209	0	0	174	174	233	247	286	304
582	90	90	105	109	128	136	217	217	0	0	176	176	233	233	307	307
583	90	90	105	109	128	136	211	217	0	0	176	196	233	253	262	298
541	90	90	105	113	130	136	213	213	0	0	176	194	235	235	274	304
534	90	90	105	113	134	136	211	217	0	0	174	196	247	247	274	307
539	90	90	105	111	130	134	209	213	0	0	176	176	245	247	274	298
590	90	90	109	111	128	136	209	209	0	0	174	176	235	245	274	286
596	90	90	109	111	130	130	207	209	0	0	174	174	233	235	298	298
552	90	90	111	111	134	134	209	217	0	0	174	176	233	247	298	298
584	90	90	105	105	128	136	211	217	0	0	176	196	233	253	262	298
595	90	107	111	113	128	136	209	211	0	0	176	176	233	249	292	314
540	90	107	105	109	130	136	217	219	0	0	174	176	233	235	298	298
